# FLIM‐FRET‐based analysis of S100A11/annexin interactions in living cells

**DOI:** 10.1002/2211-5463.13782

**Published:** 2024-02-26

**Authors:** Christian Melle, Birgit Hoffmann, Annett Wiesenburg, Christoph Biskup

**Affiliations:** ^1^ Biomolecular Photonics Group, Jena University Hospital Friedrich Schiller University Jena Germany

**Keywords:** annexin, FLIM, FRET, protein–protein interactions, S100 protein, S100A11

## Abstract

Proteins achieve their biological functions in cells by cooperation in protein complexes. In this study, we employed fluorescence lifetime imaging microscopy (FLIM)‐based Förster resonance energy transfer (FRET) measurements to investigate protein complexes comprising S100A11 and different members of the annexin (ANX) family, such as ANXA1, ANXA2, ANXA4, ANXA5, and AnxA6, in living cells. Using an S100A11 mutant without the capacity for Ca^2+^ binding, we found that Ca^2+^ binding of S100A11 is important for distinct S100A11/ANXA2 complex formation; however, ANXA1‐containing complexes were unaffected by this mutant. An increase in the intracellular calcium concentration induced calcium ionophores, which strengthened the ANXA2/S100A11 interaction. Furthermore, we were able to show that S100A11 also interacts with ANXA4 in living cells. The FLIM‐FRET approach used here can serve as a tool to analyze interactions between S100A11 and distinct annexins under physiological conditions in living cells.

## Graphical abstract

Proteins often fulfill their functions in interaction with other proteins within a protein complex. The calcium‐binding annexin proteins and S100 proteins interact with each other and are involved in cellular membrane dynamics, vesicle transport, and vesicle fusion. We have analyzed the interaction of different members of the annexin protein family with S100A10 and S100A11 using FLIM‐based FRET measurements.

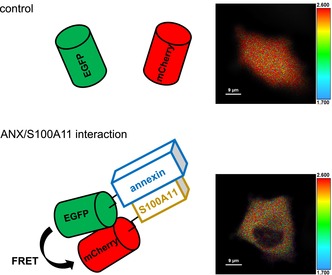

**Abbreviations**
ANX: annexin
*E*
: FRET efficiency
EGFP: enhanced green fluorescence protein
FCS: fetal calf serum
FLIM: fluorescence lifetime imaging microscopy
FP: fluorescent protein
FRET: Förster resonance energy transfer
PPI: protein–protein interaction
S100A11ΔCa: S100A11 mutant lacking Ca^2+^‐binding activity

The function and activity of a distinct protein can be modulated by interaction with other proteins. Different members of the calcium‐binding protein families annexin (ANX) and S100 can perform multiple protein–protein interactions (PPIs) with each other to promote membrane dynamics, vesicle trafficking, and vesicle fusion [1, 2, 3]. Thereby, different combinations of interacting partners occur between several S100 proteins and some annexin members to achieve their biological functions [4, 5]. A model of membrane aggregation involving a hetero‐tetrameric ANX/S100 complex has been proposed [5]. In this model, two peripherical ANX proteins interconnected by two central S100 proteins are enabled each to bind a membrane.

The main function of ANX is to hold and connect membranous structures together. Cells deficient for ANXs suffer from restricted membrane rearrangement and repair [6, 7]. Annexins are characterized by a highly conserved core domain composed of four repeat sequences (eight for ANXA6) containing alpha‐helical subdomains each [8]. Upon binding of Ca^2+^ ions by a specific loop domain localized in the C terminus, ANXs are able to bind to negatively charged phospholipids of membranes resulting in a ternary complex bridging adjacent membranes [9]. The N‐terminal domain of ANX conveying its functional specificity allows interaction with distinct partners such as members of the S100 protein family [3]. Interaction between ANXA2 and S100A11 results in binding of cytoskeleton filaments, e.g., actin, and facilitates formation of new membranes [10, 11, 12]. S100 proteins show cross‐reactivity in forming one‐to‐many interactions with annexins where, for example, S100A11 forms functional complexes with ANXA1, ANXA2, and ANXA6 [2, 13]. Since ANXA1 has been linked to structural organization of multivesicular endosomes by localizing on membranes, binding with S100A11 regulates the endosomal localization of this complex [14].

S100 proteins possess remarkable structural similarity. A unique characteristic of several S100 proteins such as S100A11 among the EF‐hand protein family is to form symmetric noncovalent homo‐dimers [15]. Binding of calcium induces a conformational change of the S100 protein resulting in exposure of a hydrophobic domain capable of interacting with partners like annexins [3]. Furthermore, Ca^2+^ triggers an S100A11/ANXA2 interaction at the site of plasma membrane repair whereas S100A11 is not involved in recruitment of ANXA1 to the membrane wound [11]. There is an S100A11ΔCa mutant described lacking the Ca^2+^‐binding domain which is suggested to be essential for Ca^2+^‐induced conformational changes [11, 16]. The interaction complex between ANXA6 and S100A11 is supposed to be involved in the Ca^2+^‐dependent linkage of the plasma membrane to the cytoskeleton [17].

Another important cellular function of S100A11 is the regulation of cell growth. Here, it plays a dual role in epithelial cells. S100A11 inhibits growth in a Ca^2+^‐depended manner by its transfer to the nucleus resulting in p21^CIP1/WAF1^ (*CDKN1A*) induction, and on the other hand, it induces growth stimulation by enhancement of the level of epidermal growth factor protein family members by its secreted form [18, 19]. Furthermore, S100A11 is regulating p21^CIP1/WAF1^ stability through the PI3K/AKT signaling pathway reflecting its capacity to inhibit apoptosis [20]. A protection against apoptosis in neuronal cells of S100A11 via preventing the nuclear translocation of ANXA1 has been recently shown [21]. S100A11 is able to modulate activities of its distinct interaction partners. As an example, S100A11 is involved in the regulation of actin cytoskeleton in a Ca^2+^‐dependent manner [22]. Furthermore, S100A11 seems to be involved in DNA damage repair as it regulates the localization and persistence of its interaction partners RAD54B and RAD51 in DNA repair foci [23, 24]. As S100A11 is a binding partner of the receptor for advanced glycation end products (RAGE), S100A11 is supposed to participate in inflammatory response [25].

It has been shown that deregulated S100A11 is involved in various human diseases including different carcinomas. Expression of S100A11 can be up‐ or down‐regulated in colorectal carcinoma, pancreatic ductal adenocarcinoma, and hepatocellular carcinoma, or bladder cancer, respectively [26, 27, 28, 29]. As S100A11 is significantly associated with high pathological stages in distinct cancer entities, deregulated S100A11 should be considered as an oncogenic factor [28, 29]. Intriguingly, there is a dualism in S100A11 expression behavior suggesting that S100A11 is also a potential tumor suppressor in distinct tumor entities as its down‐regulation correlates with development of bladder cancer [30]. Furthermore, S100A11 has been shown to be associated with metabolic diseases as well as neurological diseases [31].

To analyze PPIs between potentially interacting candidates in living cells, proteins can be labeled with fluorescent proteins, which does not only allow to trace the proteins in living cells. By exploiting Förster Resonance Energy Transfer (FRET) the molecular vicinity of the fluorescent tags and thus the interaction between labeled proteins can be assessed. FRET occurs when suitable donor and acceptor fluorophores are brought into close vicinity so that they are only separated by a distance of a few nm. In this case, the donor fluorophore can transmit its excitation energy to the acceptor fluorophore resulting in a decrease of the lifetime of the donor in the excited state, a decrease of donor fluorescence intensity and an increase of the acceptor fluorescence intensity [32]. In principle, all phenomena can be exploited to measure the efficiency of FRET (*E*). However, in contrast to intensity‐based measurements fluorescence lifetime measurements have the advantage that they are independent of the fluorophore concentration. This allows to estimate the FRET efficiency directly in living cells without the need of doing additional, sometimes error‐prone control measurements as this is the case in intensity‐based measurements of the FRET efficiency [33]. Consequently, we used in the present study fluorescence lifetime imaging microscopy (FLIM) to measure donor fluorescence lifetimes from which FRET efficiencies were calculated to assess protein interactions between S100A11 and distinct annexin (ANX) members in living cells. As label, we used green/red FP pairs, e.g., EGFP and mCherry. The emission spectrum of EGFP is well separated from that of mCherry, so that a range of donor emission wavelengths can be identified, which is devoid of contamination with acceptor fluorescence. Despite the essential separation of excitation and emission spectra, the donor emission and acceptor absorption show a high degree of spectral overlap ensuring efficient energy transfer. Moreover, green/red FPs offer greater Förster radii compared to other FP pairs allowing detection of FRET over longer distances [34].

In the present study, lifetime‐based FRET measurements were employed to assess protein interactions between S100A11 and distinct annexin (ANX) members in living cells.

## Materials and methods

### Cell culture and reagents

Human U2OS osteosarcoma cells, human HEK293T kidney carcinoma cells, and human epithelial squamous carcinoma cell line A431 were cultured in DMEM (Gibco, Paisley, UK) supplemented with 10% fetal calf serum (FCS; Sigma, Saint Louis, MO, USA). Cells were harvested at 70–90% confluence and passaged at a split ratio of 1 : 5 or, in the case of HEK293T cells, at a split ration of 1 : 10. U2OS cells, HEK293T cells, and A431 cells were obtained from the Institute of Human Genetics, Jena University Hospital (Jena, Germany).

Cells grown on glass cover slips localized in 6‐cm cell culture dish were transfected with respective expression plasmids using calcium phosphate‐based transfection. For FLIM‐FRET experiments, cells were washed 42–45 h after transfection three times with PBS to eliminate medium and covered with PBS for imaging.

To assess to which degree interaction between annexins and S100A11 can be stimulated by Ca^2+^, intracellular Ca concentrations were increased by applying a Ca^2+^ ionophore in Ca^2+^ rich solution. In brief, transfected U2OS cells were washed three times with PBS and transferred in Krebs‐Ringer buffer (145 mm NaCl, 5 mm KCl, 1.3 mm MgCl_2_, 1.2 mm NaH_2_PO_4_, 10 mm glucose, 20 mm HEPES, pH7.4) containing 3 mm CaCl_2_. Control FLIM‐FRET measurements were carried out under this condition. To analyze the effect of elevated Ca^2+^ concentrations on the PPIs, 1 μm ionomycin (ThermoFisher; Invitrogen; Eugene, OR, USA) was added and measurements of the same cells were started 1 min and 10 min after adding ionomycin.

### Plasmid construction

Human annexins (ANXA1, ANXA4, and ANXA5) were PCR amplified each from A431 cDNA using distinct oligonucleotide primers (Table 1). The PCR fragments derived from ANXA1 or ANXA5 specific primers were cloned between the *Xho*I and *Bam*HI restriction site of pEGFP‐N1 (Clontech, Mountain View, CA, USA) or pmCherry‐N1 (Clontech). The PCR fragment of ANXA4 was cloned between *Hind*III and *Bam*HI restriction site of pEGFP‐N1 or pmCherry‐N1. The AnxA6‐EGFP plasmid containing rat annexin A6 was kindly provided by C. Enrich (Universidad de Barcelona) [35]. The generation of human Annexin A2 (ANXA2) construct has been described elsewhere [36]. The human *S100A11wt* gene as well as the *S100A11ΔCa* mutant containing an eliminated Ca^2+^‐binding capacity [11] was cloned between *Eco*RI and *Bam*HI sites of pEGFP‐N1 and pmCherry‐N1. S100A10 was PCR amplified from A431 cDNA and the resulting fragment was cloned in pEGFP‐N1 or pmCherry‐N1 using *Eco*RI and *Bam*HI sites.

**Table 1:** Oligonucleotide primers for PCR amplification.

	Forward primer (5′‐3′)	Reverse primer (5′‐ 3′)
Annexin A1	actggctcgagttatggcaatggtatcagaattcc	cagtcggatccaagtttcctccacaaagagccacc
Annexin A4	actgaaagcttgtatggccatggcaaccaaaggagg	cagtggatccgaatcatctcctccacagagaacaagc
Annexin A5	cagtggatccgaatcatctcctccacagagaacaagc	cagtggatccgtgtcatcttctccacagagcagc
S100A10	acgtcagaattctatgccatctcaaatggaacacgcc	acgttggatccagcttctttcccttctgcttcatgtg
S100A11	gccccgaattcggacatggcaaaaatctccagccctaca	gtatggatcctcggtccgcttctgggaagg
mCherry (C19G)	actgaagcttatggtgagcaagggcgaggag	cagtaagcttcttgtacagctcgtccatgcc

The mCherry‐EGFP fusion construct (C19G) was used as positive FRET control. Therefore, mCherry was PCR amplified and the resulting PCR product was cloned in pEGFP‐N1 using the *Hind*III restriction site. The correct insertion of all PCR products in the distinct vectors was confirmed by sequencing.

### Fluorescence lifetime‐based FRET measurements

In this study, the physical phenomenon of Förster resonance energy transfer (FRET) was used to map protein–protein interactions of different annexins from both human and rat (ANXA1, ANXA2, ANXA4, ANXA5, and AnxA6) with human S100A11 in living U2OS or HEK293T cells. The efficiency (*E*) of energy transfer was estimated by comparing the lifetime of the donor in presence of the acceptor (*τ*_DA_) with the lifetime of the donor in absence of an acceptor (*τ*_D_) according to:

(1) $$E=1-\frac{\tau_{\mathrm{DA}}}{\tau_{D}}.$$

All lifetime measurements were done on the stage of an upright confocal laser scanning microscope (TCS SP8, Leica, Mannheim, Germany) using a 25×/0.95 HC Fluotar L water immersion objective (Leica). Before starting a FLIM experiment, confocal images were sequentially acquired which minimized bleed‐through between detection channels in order to ensure that both donor and acceptor fluorophores are present, but not overexpressed. To excite donor and acceptor a supercontinuum white light laser (SuperK Extreme, NKT Photonics, Birkerod, Denmark) was used. To excite EGFP a small band with the center at 488 nm was selected with an acousto‐optical tunable filter (AOTF) from the white light continuum, whereas a band with the center at 561 nm was chosen to excite the acceptor. Emitted fluorescence was collected in two channels. In the first channel light in the 500–550 nm range (containing almost exclusively donor fluorescence) was collected, whereas in the second channel light in the 600–650 nm range was collected, containing predominantly acceptor fluorescence. Reflected laser light was blocked with a 488 nm and a 561 nm notch filter.

For fluorescence lifetime measurements, the sample was scanned with a slow scan speed of 100 Hz and excited at 488 nm with a repetition rate of 20 MHz. EGFP fluorescence was collected in one of the confocal channels within a spectral range of 500–550 nm and detected with the in‐built hybrid detector. A directly connected time‐correlated single photon counting (TCSPC) system (HydraHarp 400, Picoquant; Berlin, Germany) was used for recording individual photon events which were saved in a continuous data stream in a time‐tagged time‐resolved, TTTR file. Photon events were collected while scanning 100 frames, corresponding to a total acquisition time of 519 s.

The recorded data were analyzed with the software of the manufacturer of the TCSPC system (symphotime 64, Version 2.2; Picoquant; Berlin, Germany). To generate fluorescence lifetime images, the decay data of 2 × 2 pixels were binned and approximated with a biexponential function. To determine the average fluorescence lifetimes in a cell, the perimeter of a cell was delineated by a region of interest (ROI) and decay data of all pixels within this region were summed up. Fluorescence decay data of single pixels as well as the fluorescence decay of a whole cell were analyzed by fitting a biexponential function to the tail of the fluorescence decay starting 200 ps after the peak. The goodness of fit was judged by the reduced Chi‐squared ($\chi_{v}^{2}$) value, and the parameters of the biexponential function were approximated by iterative reconvolution yielding a $\chi_{v}^{2}$ below 1.1. The FRET efficiency *E* was calculated using Eqn (1) [37].

A single measurement of a sample was considered significantly different from a control measurement of the donor only sample when its mean fluorescence lifetime differed from the mean of the control fluorescence lifetime by more than three standard deviations (SD). Thus, FRET was assumed to occur when the measured fluorescence lifetime of a sample cell was shorter by more than three standard derivations (SD) of the control measurements that were carried out in absence of an acceptor.

Amplitude‐weighted mean lifetimes (τ_m,x_) measured in cells (τ_m,c_) were calculated according to:

(2) $$\tau_{m,x=A_{f}\tau_{f}+A_{s}\tau_{s}.}$$

where *A*_f_ and *A*_s_ are the amplitudes of the fast (*τ*_f_) and slow (*τ*_s_) decay components, respectively.

Average lifetimes were obtained from the indicated number of cells in three independent experiments and are presented as an average of the amplitude‐weighted mean lifetimes ± standard error of the mean (SEM). In the fluorescence lifetime images, lifetimes are encoded by color Red represents long lifetimes (2.60 ns), whereas blue encodes short lifetimes (1.70 ns).

Control experiments were done to ensure that FRET can be reliably detected with the experimental setup. First, untransfected cells were measured to estimate the amount of autofluorescence background. The contribution of autofluorescence was negligible (< 0.5%) at the excitation wavelength and excitation intensity used throughout the FLIM measurements. As negative FRET control, U2OS or HEK293T cells co‐expressing unfused EGFP and mCherry were used to exclude that non‐specific FRET occurred because of molecular crowding or non‐specific interaction of the fluorophores themselves.

For validation of the TCSPC instrumentation, we used coumarin‐6 (100 μm; in EtOH) as fluorescence lifetime standard. The lifetime of coumarin‐6 under these conditions at room temperature was 2.49 ns which was comparable to published data [38]. Before each measurement, the lifetime was determined to ensure that the system was properly adjusted and calibrated. Since the time course of the fluorescence decay deviated from that of reference measurements during the first 200 ps after the laser pulse, only the tail of the fluorescence decay was analyzed.

### Statistical analysis

Each experiment was repeated at least three times. Data are presented as mean ± SEM. Statistical comparisons were performed using two‐sided unpaired *t*‐test for non‐parametric data sets. A *P* value of < 0.05 was considered statistically significant.

## Results

### Validation of FLIM‐FRET for protein–protein interaction analysis in living cells

**Fig. 1:**
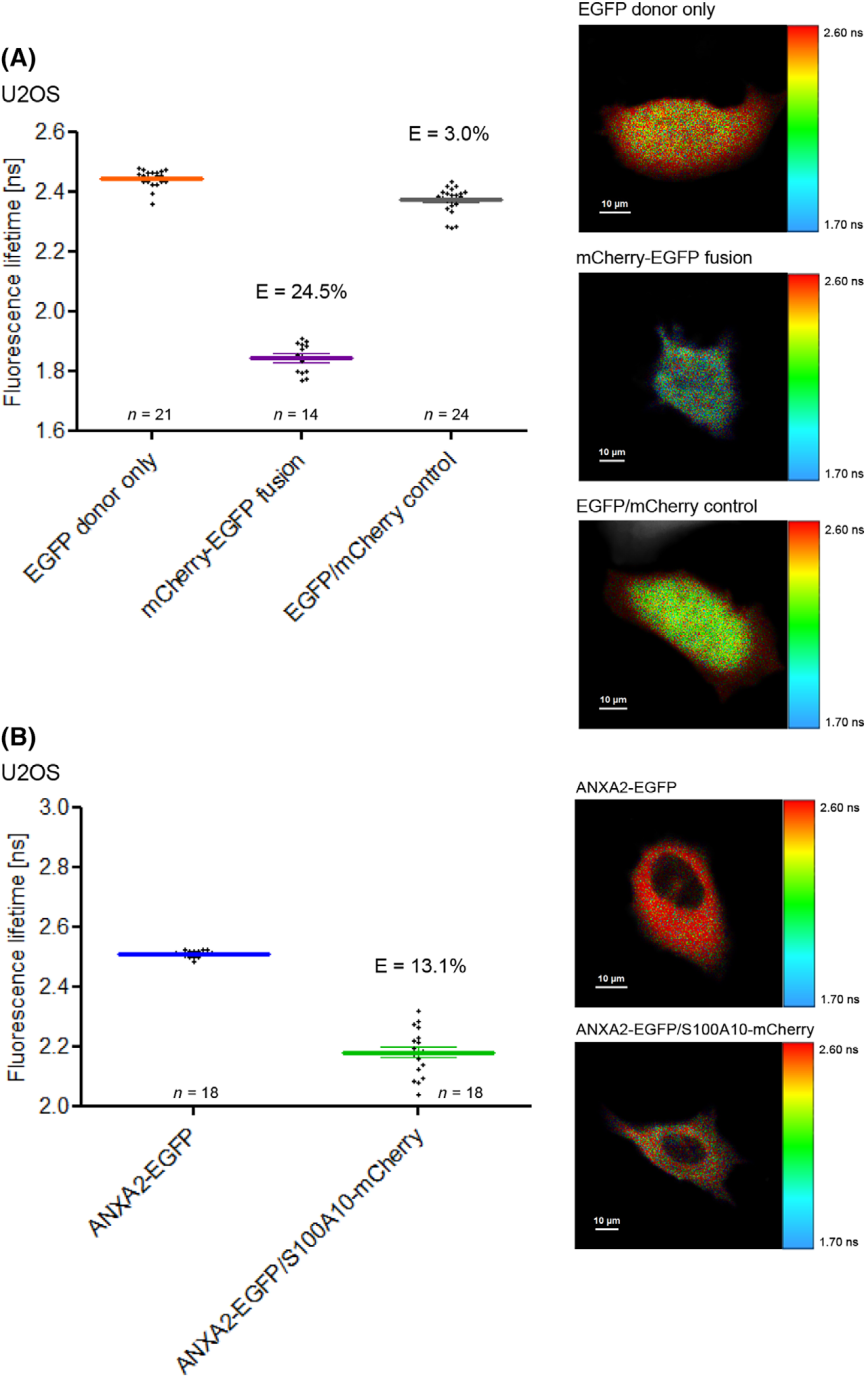
Validation of the FLIM‐FRET analysis of protein–protein interactions in living U2OS cells. (A) *Left panel*: Fluorescence lifetimes [ns] and corresponding FRET efficiencies (*E*) were determined in U2OS cells expressing EGFP only, the mCherry‐EGFP fusion protein, or unfused EGFP and mCherry. Error bars show mean and SEM of amplitude‐weighted mean fluorescence lifetimes obtained from the indicated number of measurements in three independent experiments. *Right panel*: Fluorescence lifetime images of representative cells. Fluorescence lifetimes are encoded by color ranging from blue (corresponding to 1.70 ns) to red (corresponding to 2.60 ns). (B) Analysis of protein–protein interaction between ANXA2 and S100A10. *Left panel*: Fluorescence lifetimes of EGFP measured in cells expressing only ANXA2‐EGFP or in cells co‐expressing ANXA2‐EGFP/S100A10‐mCherry. Error bars show mean + SEM of amplitude‐weighted mean fluorescence lifetimes obtained in the indicated number of cells in three independent experiments. Corresponding FRET efficiencies (*E*) are indicated. *Right panel* Fluorescence lifetime images of representative cells with the same color scale as in A. are presented as color‐coded representation (1.70–2.60 ns). Scale bars are indicated in the respective images.

**Fig. 2:**
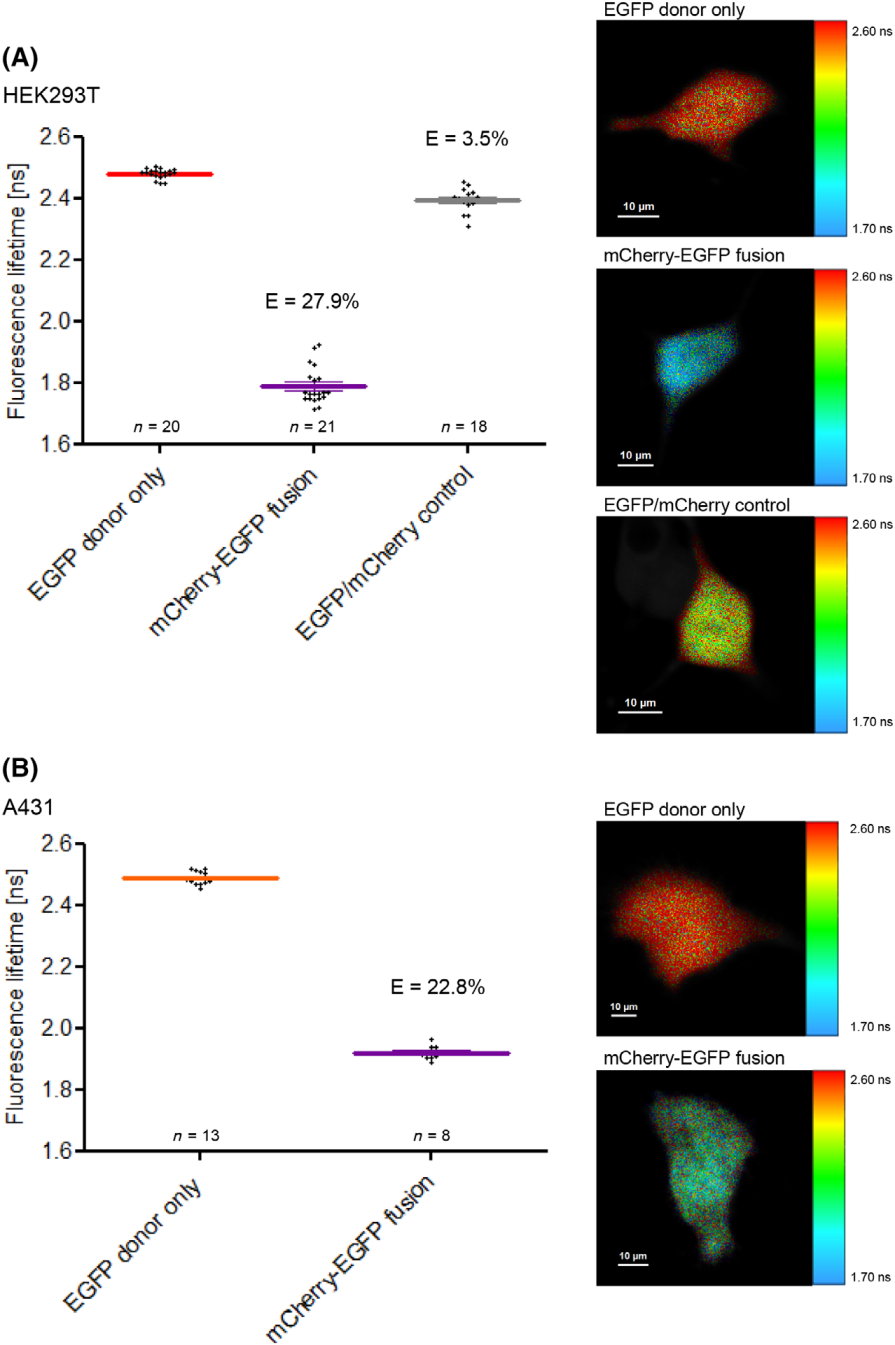
Fluorescence lifetimes and FRET efficiencies (*E*) of EGFP, the mCherry‐EGFP fusion protein and EGFP/cCherry expressed in HEK293T cells (A) or A431 cells (B). *Left panel*: Fluorescence lifetimes and corresponding FRET efficiencies. *Right panel*: Fluorescence lifetime images of representative cells. Fluorescence lifetimes were encoded in the same way as described in Fig. 1. Error bars show mean and SEM of amplitude‐weighted mean lifetimes measured in the indicated number of cells in three independent experiments. Scale bars are indicated in the respective images.

As S100A11 is able to interact with several distinct members of the annexin family as shown *in vitro* by biochemical approaches [17, 39, 40, 41], we tried to find out whether these protein–protein interactions (PPIs) can be analyzed directly in living cells by employing fluorescence lifetime imaging microscopy (FLIM) to test if FRET occurred between the labeled proteins. To validate our experimental procedure, we generated a fusion construct comprising mCherry and EGFP linked by a 19 amino acid peptide (C19G) that served as positive FLIM‐FRET control (Fig. 1A). A fusion construct containing a donor and an acceptor fluorophore connected by a peptide linker is commonly used to assess FLIM‐FRET approaches [42]. As compared to EGFP alone, which has a lifetime of 2.44 ns, the lifetime of EGFP decreased to 1.84 ns in our mCherry‐EGFP fusion expressed in U2OS cells. According to Eqn (1) this corresponds to a FRET efficiency of approx. 25% which is in the same magnitude as comparable fusion constructs [34, 43]. Comparable FRET efficiencies were detected when we expressed the mCherry‐EGFP fusion construct in HEK293T cells or in A431 cells showing a FRET efficiency of 28% and 23%, respectively (Fig. 2). This shows that the FRET efficiency is independent of the kind of cell and just determined by the fusion construct itself.

As a negative control, we also co‐expressed unfused EGFP and mCherry in cells. Here, the donor lifetime is only slightly lower than the donor only control. This excludes that at the expression levels we used in our experiments FRET efficiencies beyond 3% are caused solely by molecular crowding or by an interaction of the fluorescent tags.

To further test our experimental design, we analyzed the already established protein–protein interaction (PPI) between annexin A2 (ANXA2) and S100A10 (Fig. 1B). S100A10 exists in the cell as a tight interaction partner of ANXA2. It is rapidly degraded in the absence of ANXA2 [44]. ANXA2 and S100A10 seem to be involved in F‐actin bundling but must exist together in a complex for this function [36, 45]. In contrast to fluorescent proteins expressed as single proteins or as fusion that were distributed in both cytoplasm and nucleoplasm, ANXA2 and S100A10 were mostly detectable in the cytoplasm as shown in Fig. 1B [46]. From this, we can conclude that the fluorescent protein – tag has no influence on cellular distribution of the analyzed proteins in our experiments. We determined a FRET efficiency (*E*) of 13.1% in cells co‐expressing ANXA2‐EGFP and S100A10‐mCherry (Fig. 1B), confirming that the used FLIM‐FRET approach was appropriate to detect S100A10/annexin interactions in living cells.

### S100A11 Ca^2+^‐binding capacity is critical for interaction with specific annexin

**Fig. 3:**
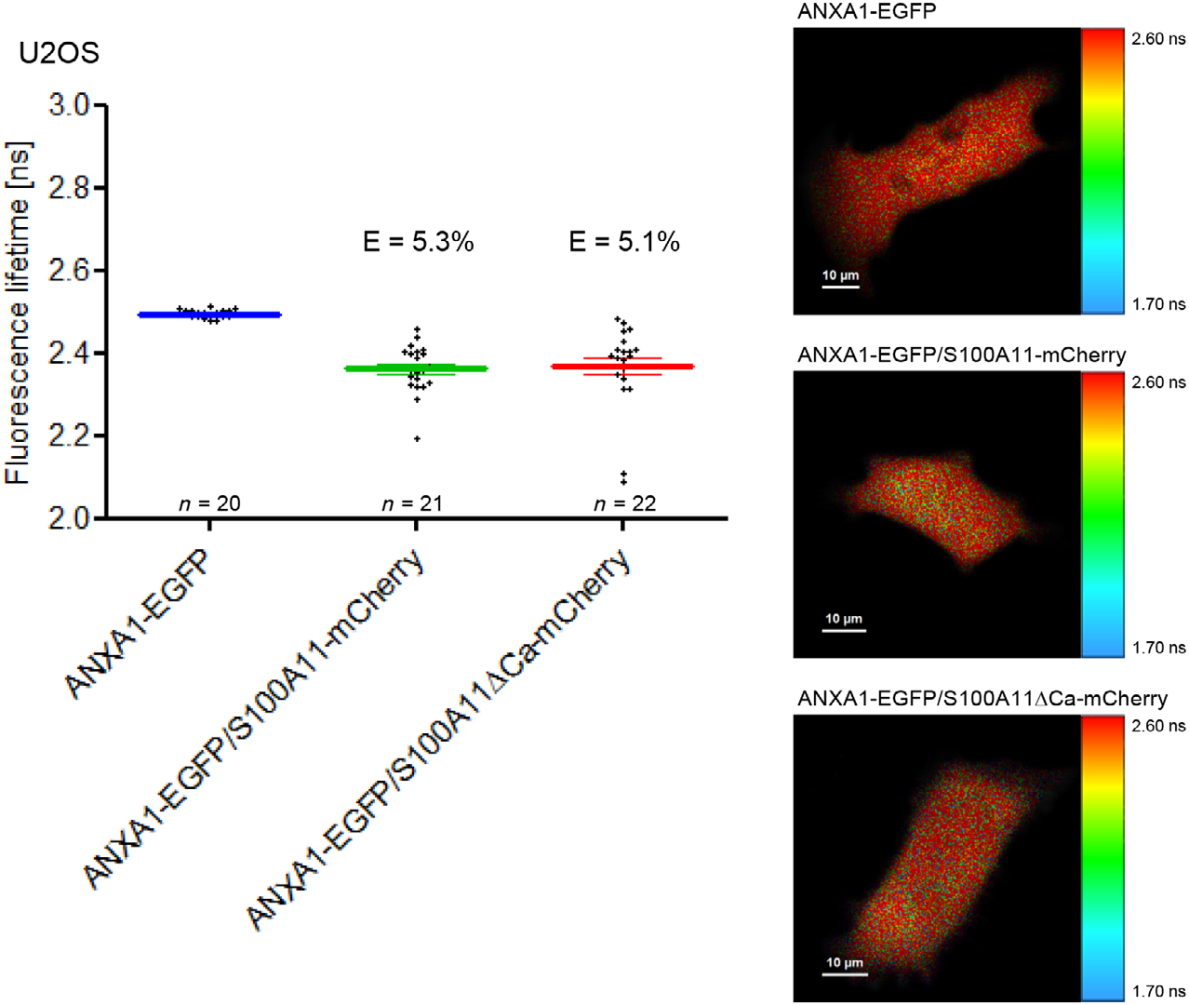
Analysis of protein–protein interaction between S100A11 and ANXA1 by FLIM‐FRET. *Left panel* EGFP fluorescence lifetimes obtained in U2OS cells transfected with ANXA1‐EGFP alone, ANXA1‐EGFP/S100A11‐mCherry, or ANXA1‐EGFP and S100A11ΔCa‐mCherry. Error bars show mean and SEM of amplitude‐weighted mean lifetimes measured in the indicated number of cells in three independent experiments. Corresponding FRET efficiencies (*E*) are indicated. *Right panel* Fluorescence lifetime images of representative cells. Scale bars are indicated in the respective images.

S100A11 occurs as a homo‐dimer in cells, which is its functional status and a prerequisite for interaction with other proteins [15]. Homo‐dimerization of S100A11 is triggered by Ca^2+^‐binding and therefore required for interaction with its partners [16, 47]. When we assessed the interaction between S100A11 and ANXA1 in living cells by FLIM‐FRET, we detected a reduction of the donor lifetime in cells co‐expressing ANXA1‐EGFP and S100A11‐mCherry compared to cells expressing ANXA1‐EGFP only suggesting an interaction between ANXA1 and S100A11 characterized by a FRET efficiency of 5.3% (Fig. 3). As Ca^2+^‐binding of S100A11 has been proposed to be essential for binding of partners [15], we next analyzed an S100A11 mutant lacking Ca^2+^‐binding activity (S100A11ΔCa) [11, 24]. In this S100A11 mutant, the calcium affinity is decreased by only few point mutations in the Ca^2+^‐binding domain [11]. It can be assumed that these point mutations will most likely have no major effect on the 3D structure of the protein. Using the S100A11ΔCa mutant, we detected only slight differences regarding lifetime of EGFP in cells expressing the mutant form of S100A11 compared to the wild‐type form. Furthermore, FRET efficiencies in experiments using wild‐type or S100A11 Ca^2+^‐binding mutant were comparable (Fig. 3). Hence, binding of Ca^2+^ by S100A11 seems to be without an impact on its protein–protein interaction with ANXA1.

**Fig. 4:**
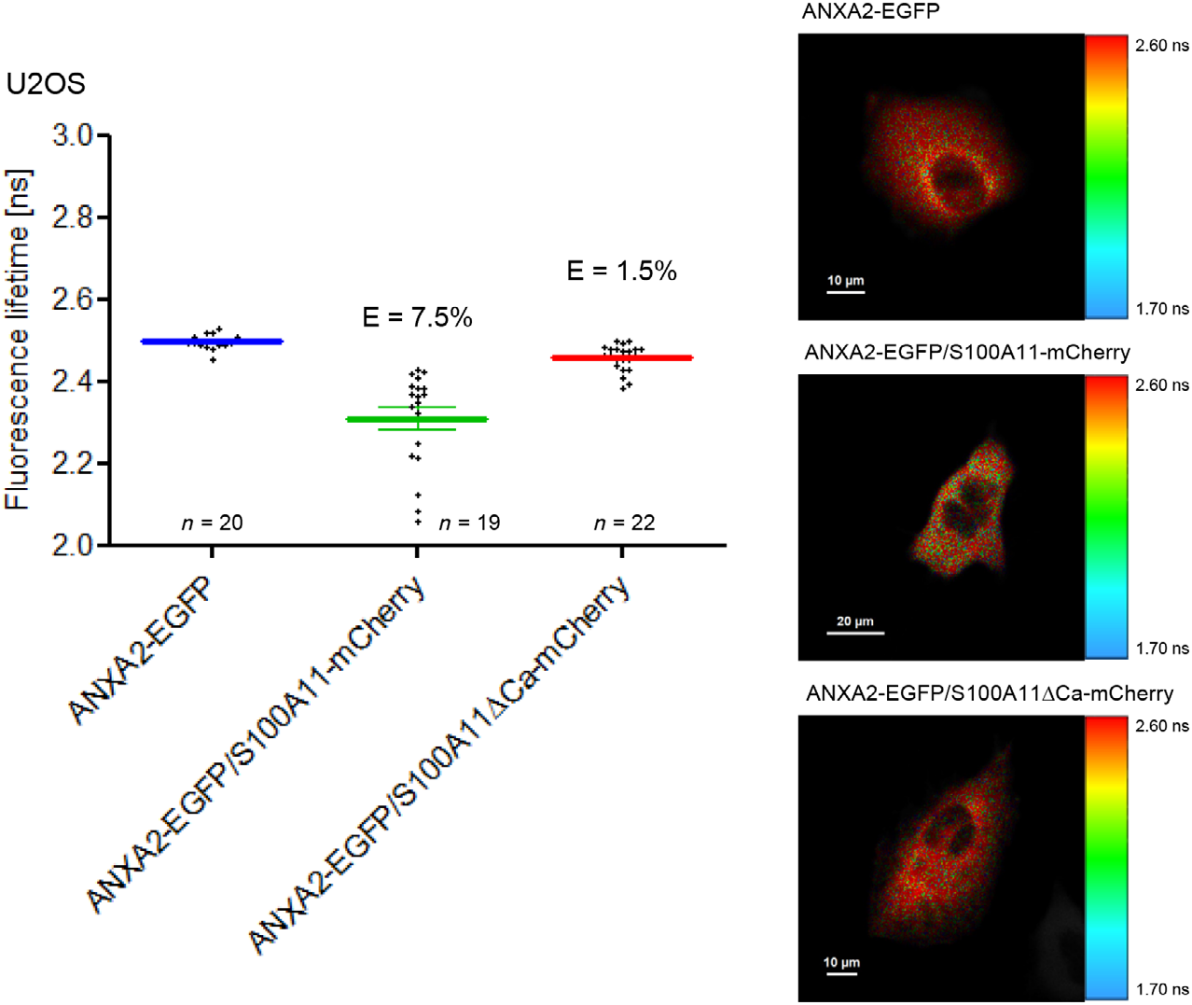
Ca^2+^‐binding to S100A11 is critical for protein–protein interaction with ANXA2. *Left panel* U2OS cells co‐transfected with wild‐type S100A11‐mCherry and ANXA2‐EGFP exhibit a smaller lifetime and higher FRET efficiency than cells co‐expressingS100A11ΔCa‐mCherry and ANXA2‐EGFP. Error bars show mean and SEM of amplitude‐weighted mean fluorescence lifetimes obtained in the indicated number of measurements in three independent experiments. *Right panel* Fluorescence lifetime images of representative cells. Scale bars are indicated in the respective images.

**Fig. 5:**
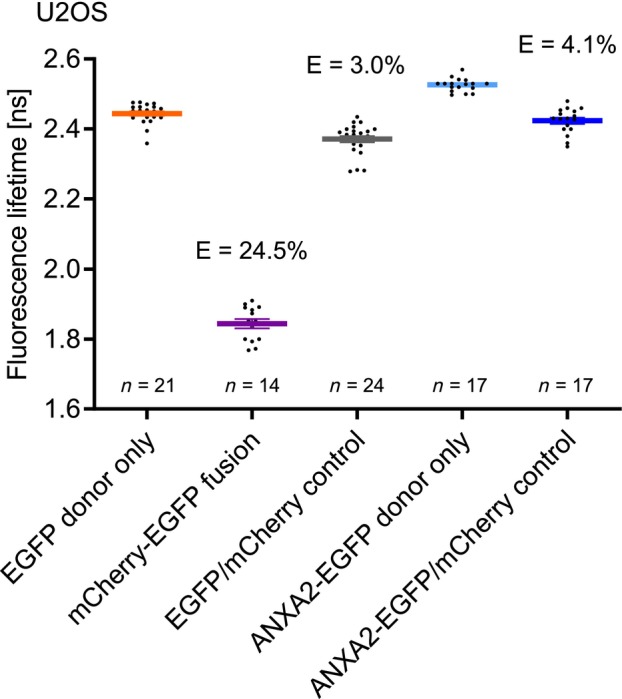
Fluorescence lifetimes and FRET efficiencies (*E*) obtained in U2OS cells expressing EGFP, the mCherry‐EGFP fusion protein, EGFP and mCherry, ANXA2‐EGFP, and ANXA2‐EGFP/mCherry (Data showing the EGFP donor only lifetime, the mCherry‐EGFP fusion, and EGFP/mCherry control are the same as shown in Fig. 1A). Scale bars are indicated in the respective images.

The analysis of S100A11/ANXA2 interaction by FLIM‐FRET showed a significant reduction of EGFP lifetime in cells co‐expressing ANXA2‐EGFP and S100A11‐mCherry compared to cells expressing ANXA2‐EGFP alone (Fig. 4). Here, we determined a FRET efficiency of 7.5%. When we examined the S100A11ΔCa mutant, EGFP fluorescence lifetime was close to the donor only control (ANXA2‐EGFP alone) resulting in a decreased FRET efficiency of 1.5% that reflects a lack of specific interaction. Hence, the Ca^2+^‐binding capacity of S100A11 seems to be critical for the S100A11/ANXA2 interaction in living U2OS cells (Fig. 4). When ANXA2‐EGFP was expressed together with an unfused mCherry as further control, the resulting FRET efficiency was comparable to the FRET efficiency obtained in EGFP/mCherry control experiments (Fig. 5).

**Fig. 6:**
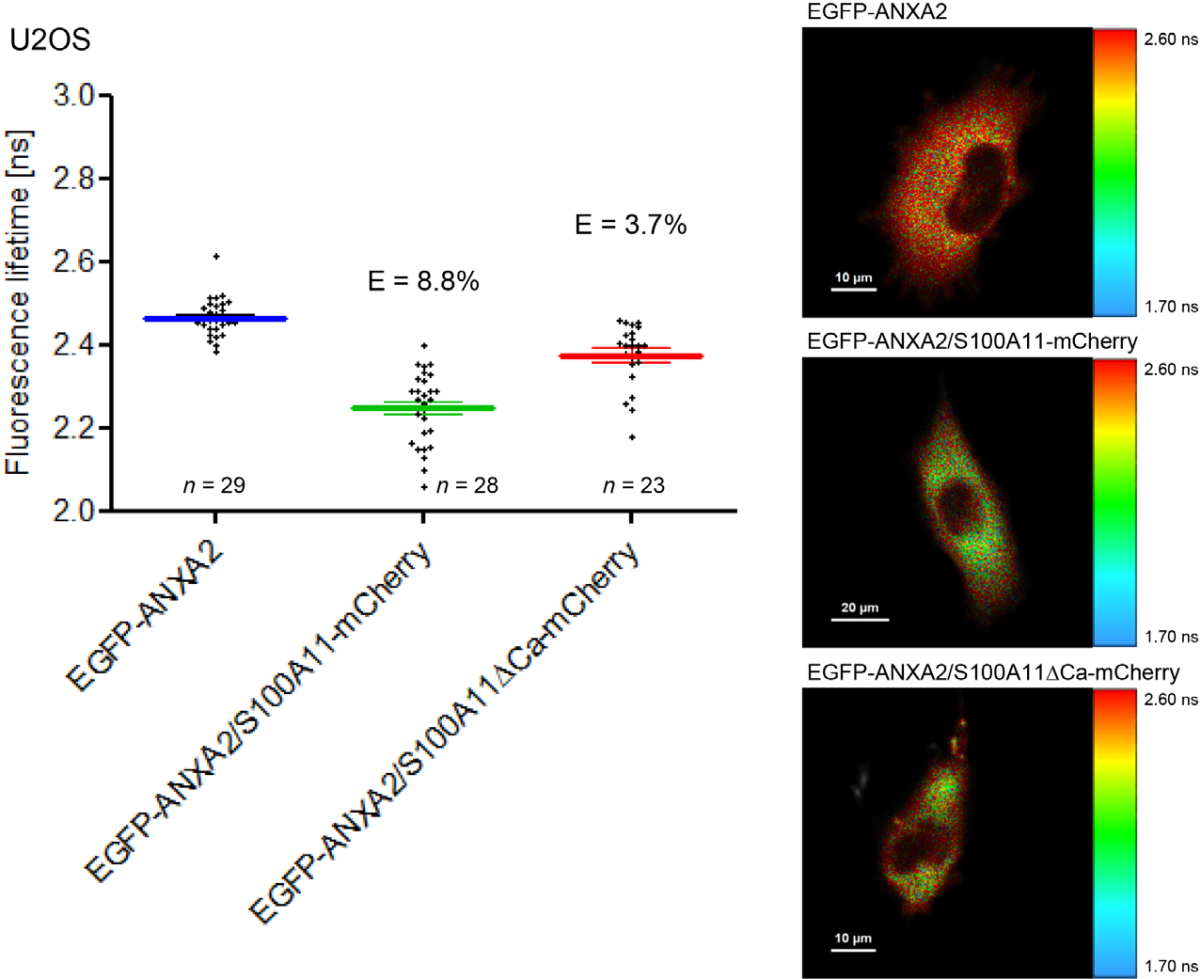
The site of attachment of the FP – tag has little impact on measured FRET efficiencies. *Left panel* EGFP fluorescence lifetimes measured in U2OS cells expressing only EGFP‐ANXA2, EGFP‐ANXA2/S100A11‐mCherry, or EGFP‐ANXA2/S100A11ΔCa‐mCherry. *Right panel* Fluorescence lifetime images of representative cells. Scale bars are indicated in the respective images.

**Fig. 7:**
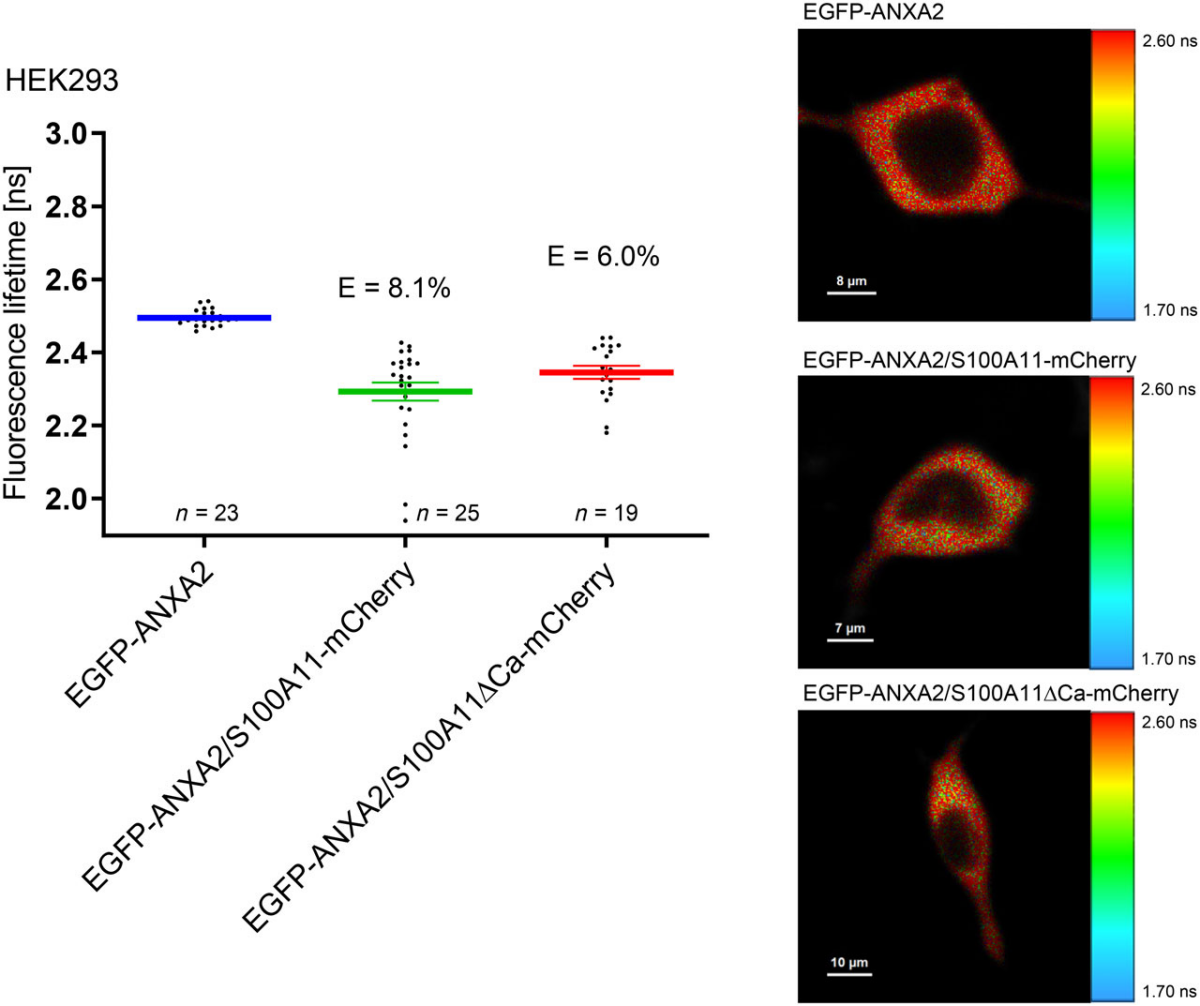
HEK293T cells expressing EGFP‐ANXA2 only, co‐expressing EGFP‐ANXA2/S100A11‐mCherry, or or EGFP‐ANXA2/S100A11ΔCa‐mCherry. *Left panel* Donor fluorescence lifetimes and FRET efficiencies. *Right panel* Fluorescence lifetime images of representative cells. Scale bars are indicated in the respective images.

To further assess the S100A11/ANXA2 interaction, we altered the orientation of the FP – tag on ANXA2 generating EGFP‐ANXA2 (Fig. 6). U2OS cells co‐expressing ANXA2 and S100A11 showed a FRET efficiency of nearly 9% indicating a specific interaction. In this case, the location of the fluorescent tag has no measurable influence on the extent of energy transfer. Analyzing the S100A11ΔCa mutant, the low FRET efficiency of 3.7% suggests a weak S100A11/ANXA2 association. Comparable results have been detected in HEK293T cells also showing decreased FRET efficiencies in cells expressing the S100A11 mutant compared to cells expressing S100A11 wild‐type (Fig. 7).

**Fig. 8:**
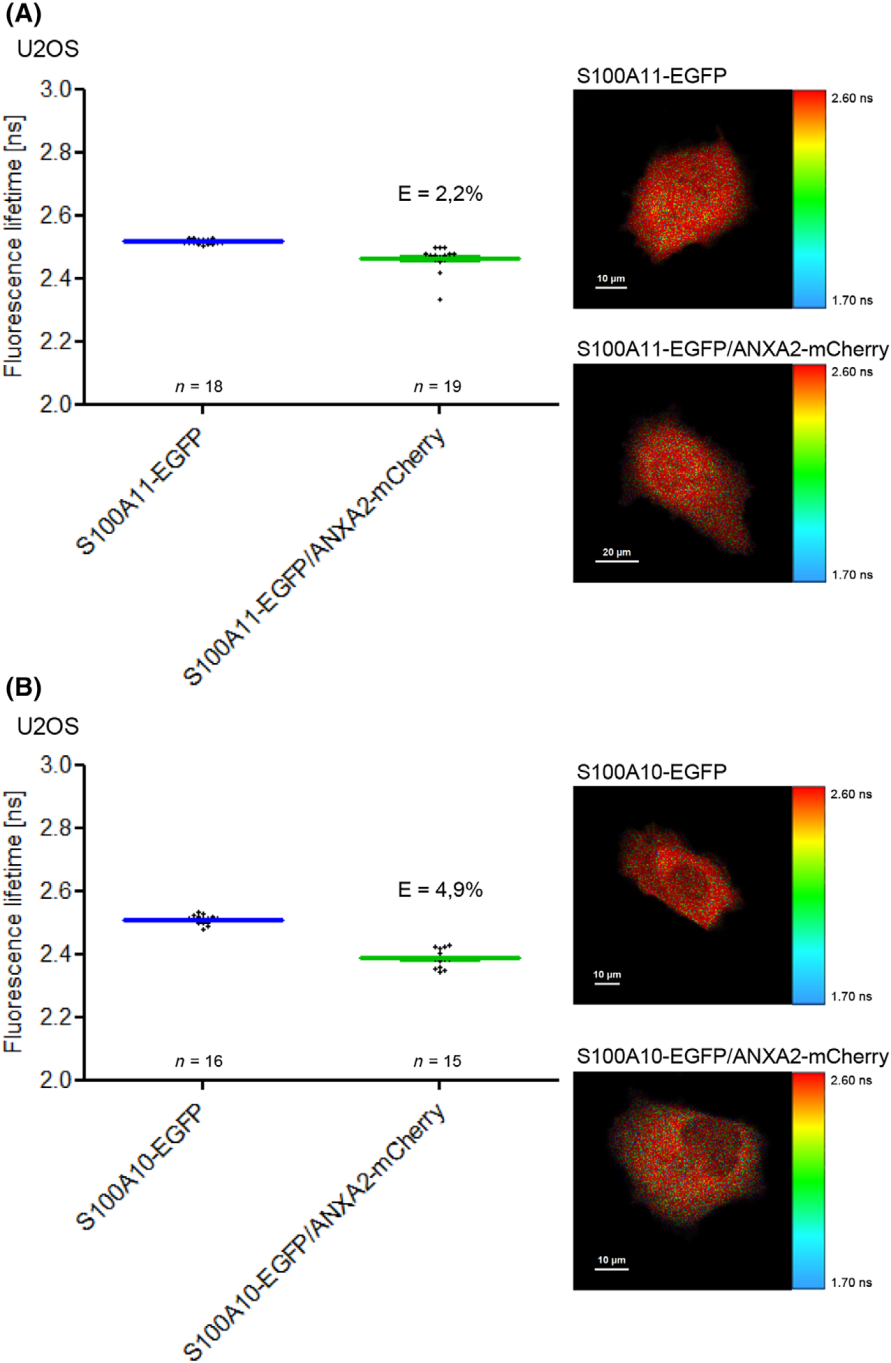
Effects of swapping the fluorescent tags. (A) Fluorescence lifetimes and FRET efficiencies measured in U2OS cells expressing S100A11‐EGFP alone or S100A11‐EGFP/ANXA2‐mCherry. (B) Fluorescence lifetimes and FRET efficiencies measured in cells expressing S100A10‐EGFP alone and S100A10‐EGFP/ANXA2‐mCherry. Scale bars are indicated in the respective images.

Furthermore, we were interested whether FLIM‐FRET measurements are influenced by swapping the donor and acceptor tags. In contrast to experiments where ANXA2 was labeled with the donor fluorophore, in approaches where S100A11 was tagged with EGFP, we detected only a slight decrease of EGFP fluorescence lifetime that results in a weak FRET efficiency (Fig. 8A). To verify these results, we analyzed the established ANXA2/S100A10 complex but used S100A10‐EGFP as donor (Fig. 8B). Again, we determined an EGFP lifetime that was significantly higher compared to measurements carried out with ANXA2‐EGFP as donor (Fig. 1B). The higher FRET efficiency in case of ANXA2 labeled with the donor fluorophore might be attributed to differences in donor to acceptor stoichiometry within the complexes due to the homo‐dimeric structure of S100A11. Another explanation could be that FRET might be hampered due to unfavorable dipole orientations of the swapped donor and acceptor fluorophore tags.

### Analysis of further S100A11/annexin interactions by FLIM‐FRET

**Fig. 9:**
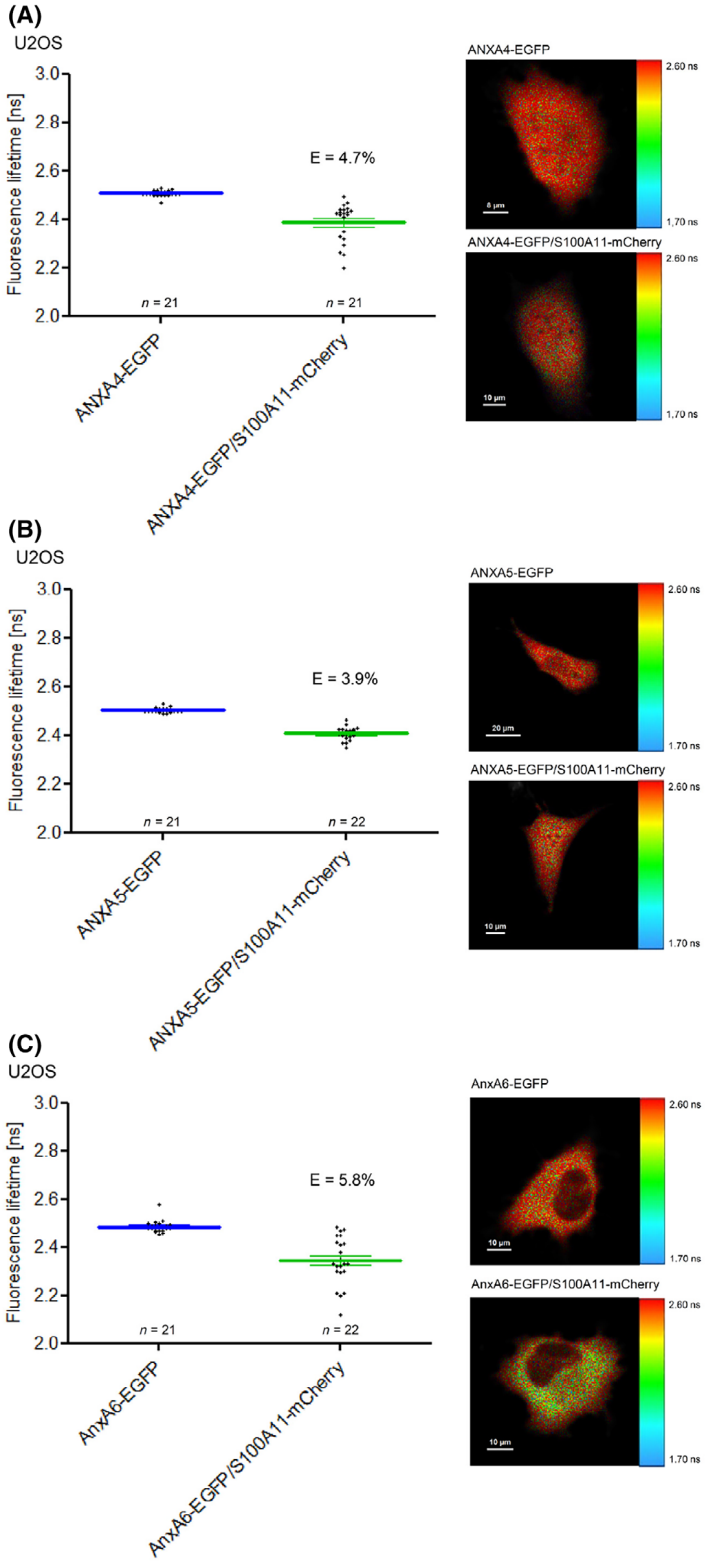
Analysis of protein–protein interactions between S100A11 and specific members of the annexin family by FLIM‐FRET. Analysis of complex formation of human S100A11 and human ANXA4 (A), human ANXA5 (B), or rat AnxA6 (C). *Left panels* EGFP fluorescence lifetimes (mean + SEM) obtained in the indicated number of cells in three independent experiments indicating potential protein–protein interactions. *Right panel* Lifetimes of representative cells are presented as color‐coded representation. Scale bars are indicated in the respective images.

We used our FLIM‐FRET approach to assess protein–protein interactions of S100A11 with further annexin family members. The measurement of ANXA4‐EGFP and S100A11‐mCherry resulted in a small but significant decrease of EGFP fluorescence lifetime compared to U2OS cells expressing ANXA4‐EGFP alone. The corresponding FRET efficiency of nearly 5% suggests an weak interaction between S100A11 and ANXA4 in living cells which to our knowledge was not reported so far in the literature (Fig. 9A). When we analyzed ANXA5‐EGFP and S100A11‐mCherry in U2OS cells, only a low FRET efficiency of 3.9% was detectable that might reflect a weak PPI or might have been caused by random collision (Fig. 9B). Furthermore, we examined the S100A11 interaction with rat AnxA6 instead of human ANXA6 by our FLIM‐FRET approach as we could not generate an ANXA6 containing vector using cDNA derived from human cell lines A431 and HaCaT, respectively. Here, a clear reduction of EGFP fluorescence lifetime was detected in U2OS cells co‐expressing AnxA6‐EGFP and S100A11‐mCherry compared to AnxA6‐EGFP only expressing cells. The corresponding FRET efficiency of approx. 6% suggests a distinct S100A11/AnxA6 protein complex (Fig. 9C).

### Increasing intracellular Ca^2+^ concentrations increase FRET efficiencies between ANXA2‐EGFP and S100A11‐mCherry interaction

**Fig. 10:**
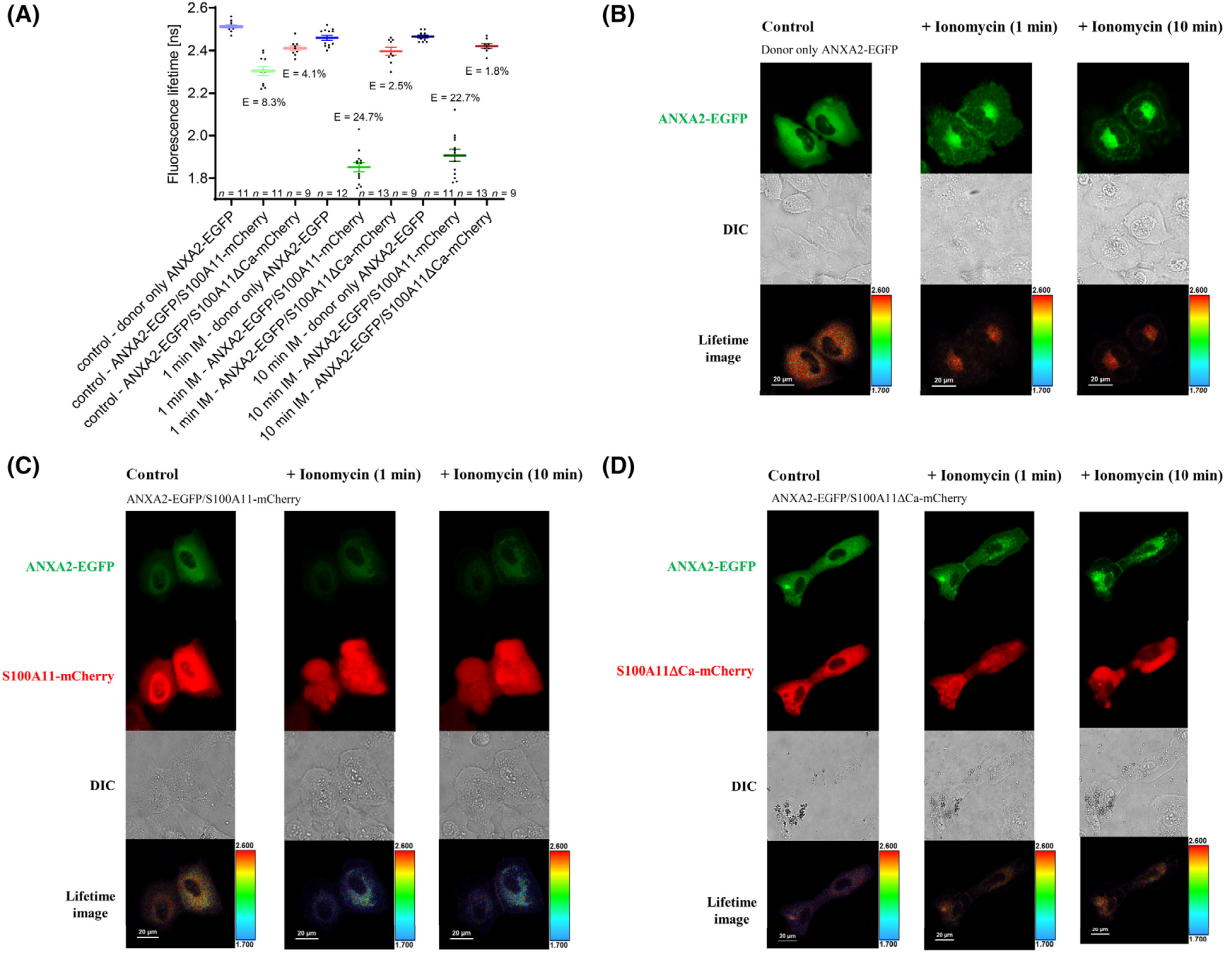
Elevated intracellular calcium concentrations increase FRET efficiencies measured in ANXA2‐EGFP/S100A11‐mCherry and ANXA2‐EGFP/S100A11ΔCa‐mCherry expressing cells. (A) Analysis of EGFP lifetimes of ANXA2‐EGFP only (blue), ANXA2‐EGFP/S100A11‐mCherry (green), and ANXA2‐EGFP/S100A11ΔCa‐mCherry (red) in untreated living U2OS cells, 1–9 and 10–18 min, after addition of ionomycin (IM). (B–D) Confocal, transmission, and fluorescence lifetime images of representative cells. Scale bars are indicated in the respective images.

Since we are mainly interested in the measurement of annexin/S100 protein–protein interactions under normal physiological conditions in living cells, all experiments discussed so far were performed in native cells at normal cytosolic calcium concentrations. As both annexins and S100A11 are Ca^2+^‐binding proteins, we tested if ANXA2/S100A11 PPI can be further stimulated by elevated intracellular calcium concentrations. To this aim, we applied the ionophore ionomycin to cells immersed in calcium‐rich extracellular solution. Here, we calculated a FRET efficiency of 8.3% from the lifetimes obtained from U2OS cells expressing the ANXA2‐EGFP donor only and from the lifetimes obtained from U2OS cells overexpressing ANXA2‐EGFP and S100A11‐mCherry. The FRET efficiencies determined here and in experiments discussed above (Fig. 4) are comparable. When we artificially raised the calcium intracellular level using Ca^2+^‐containing extracellular medium and ionomycin, a Ca^2+^ ionophore [48], a significant increase of FRET efficiency (*E*) was already detectable 1–9 min after addition of 1 μm ionomycin (Fig. 10). As the time needed for a FRET measurement is approx. 9 min, ionomycin was incubated 10 min in total. The additional incubation of ionomycin for further 10 min did not generate an additional increase in FRET efficiency (*E*). The analysis of the S100A11 Ca^2+^‐binding mutant (S100A11ΔCa‐mCherry) showed an increased FRET efficiency, but this increase was considerable lower compared to the FRET efficiency (*E*) derived from wild‐type S100A11 (Fig. 10A). The non‐physiological increase of the intracellular Ca^2+^ concentration resulted also in an obvious alteration of U2OS cell shape (Fig. 10B).

## Discussion

In the present study, we provide data about protein–protein interactions (PPIs) of S100A11 and members of the annexin (ANX) family in living cells using a fluorescence lifetime‐based FRET measurements. Our FLIM measurements allowed to calculate the FRET efficiency (*E*) which in turn provides the possibility to assess PPIs between human S100A11 and distinct annexins derived from humans or rats. FLIM‐FRET measurements benefit from the fact that fluorescence lifetimes are in contrast to fluorescence intensities independent of the fluorophore concentration. Fluorescence lifetimes are a property of a fluorophore in its specific environment, which in our experiments is determined by the interaction partners.

To determine the FRET efficiency, only the quenched lifetime of the donor in presence of the acceptor needs to be compared with the lifetime in absence of the acceptor (see Eqn 1). Since lifetimes are a property of the interacting and non‐interacting protein, both measurements can be performed in different cells.

In contrast to this, in intensity‐based measurement, the intensities in presence or absence of the acceptor must be determined in the same cell, since the measured intensities depend on the expression level of the respective fluorescently labeled proteins. Whereas the intensity of the donor in presence of the acceptor is directly accessible, the intensity of the donor in absence of the acceptor can only be measured after photobleaching of the acceptor or by estimating from the sensitized acceptor fluorescence the loss of fluorescence which is due to FRET. The first intensity‐based approach has the disadvantage that FRET efficiency can only be determined once. The second approach has the disadvantage that fluorescence recorded in the acceptor channel is not only due to sensitized acceptor fluorescence, but often contaminated by directly excited acceptor fluorescence and overlapping donor fluorescence. To correct for these contaminations, additional error‐prone control measurements are necessary [33]. In the FLIM measurements presented here, the problem of cross‐talk does not exist, since always a region of donor fluorescence can be identified which is devoid of acceptor fluorescence. For our measurements, we decided to choose the wavelength range of 500–550 nm. At 550 nm mCherry fluorescence is below 0.1% of the mCherry fluorescence at the emission maximum. Since acceptor fluorescence is not evaluated, cross‐excitation of the acceptor, which in case of the EGFP/mCherry FRET pair is not negligible, is not a problem. The selected EGFP and mCherry fluorophores are commonly used in FRET experiments [42, 49]. Furthermore, mCherry has as opposed to many other red fluorescent proteins the advantage that it matures fast [34, 50]. The FRET efficiencies measured for our mCherry‐EGFP (C19G) fusion construct used as positive FRET control were in line with results obtained for fusion constructs using other green/red FP pairs [34, 43].

In living cells, protein–protein interactions give rise to two lifetime components. The short lifetime component can be attributed to interacting proteins. Here, the lifetime of the doner tag is quenched due to FRET. In contrast to this, the long lifetime component originates from free, non‐associated proteins, whose donor tag is not quenched by FRET. In this study we determined the amplitude‐weighted mean lifetime (see Eqn 2) to assess if FRET occurs. The amplitude‐weighted mean lifetime depends not only on the lifetimes of the associated and non‐associated donor fluorophores, but also their relative fraction. Since FRET efficiencies presented in this study were calculated from the amplitude‐weighted lifetimes, they also depend on the individual lifetimes and their amplitudes.

So far, several protein complexes between S100A11 and distinct annexin family members such as ANXA1, ANXA2, and ANXA6 were characterized by biochemical approaches like pull‐down and co‐immunoprecipitation [2, 3]. Annexins as well as S100A11 are both able to bind Ca^2+^ that induces conformational changes. S100A11 is in cells as homo‐dimer present which is its functional state. Homo‐dimerization is induced by Ca^2+^‐binding of S100A11 [15, 16, 51]. Only the homo‐dimeric form of S100A11 is supposed to interact with annexins [2, 3]. In addition to the wild‐type S100A11, we examined also an S100A11 mutant, in which Ca^2+^‐binding capacity was eliminated by mutating aspartic acid and glutamic acid residues in the Ca^2+^‐binding motifs to serine [11]. In contrast to previous work demonstrating the impact of Ca^2+^ on the interaction between ANXA1 and S100A11 [14, 39], we detected almost the same FRET efficiency between EGFP labeled human ANXA1 and the mCherry labeled S100A11 wild‐type or the S100A11ΔCa mutant ectopically expressed in U2OS cells (Fig. 3). Hence, Ca^2+^‐binding of S100A11 seems to be less important for interaction with ANXA1 in our expression system. These different findings can be explained by the fact that the S100A11ΔCa mutant lacking Ca^2+^‐binding capacity used in the present study is different from the S100A11D91stop mutant lacking residues 91–99 which contain the ANXA1‐binding site [52].

Comparison of the FRET efficiency observed between EGFP labeled human ANXA2 and the mCherry labeled S100A11 wild‐type (7.5%) or the S100A11ΔCa mutant (1.5%) show that S100A11/ANXA2 interaction depends on Ca^2+^ induced conformational changes of S100A11 (Fig. 4). This finding is consistent with a previous study [41]. An unexpected observation for that we have no completely cogent explanation must be mentioned regarding the lower FRET efficiency (1.5%) of the mutant S100A11 and ANXA2 pair (Fig. 4) as compared to (3%) seen for the non‐specific control FRET between free EGFP and mCherry (Fig. 1). The fact that the FRET efficiency determined for EGFP and mCherry is higher than the efficiency determined in cells co‐expressing ANXA2‐EGFP and S100A11∆Ca‐mCherry might be caused by the fact that the unfused tags have a lower molecular weight than the tags fused to proteins. They diffuse faster and are more likely to come into close distance of an acceptor during their excited state lifetime.

The increase of the intracellular calcium concentration caused by application of the ionophore ionomycin in a Ca^2+^‐rich extracellular solution caused an increase of the FRET efficiency to 24.7% (Fig. 10) suggesting that more ANXA2/S100A11 complexes were formed. Here, however, it must be noted that an alteration of cell's shape was obviously. Furthermore, the localization of the FP‐tagged proteins in the cell was changed after addition of ionomycin which might have affected the FRET efficiency (*E*), too. In contrast to Jaiswal et al. who showed an S100A11ΔCa/ANXA2 interaction by co‐immunoprecipitation [11], we did not detect an interaction between the S100A11ΔCa mutant and ANXA2 in U2OS cells using our FLIM‐FRET approach. As an explanation for the differences between our data and the data reported by others, it is important to consider that each of assessed interactions may behave differently in different expression systems or as analyzed by different approaches. It was described that S100A11 and ANXA2 modulate plasma membrane repair by mutually dependent co‐accumulation of the complex at membrane damage sites in which the S100A11/ANXA2 accumulation is independent of ANXA1 [11]. Very recently, an ANX‐independent role of S100A11 in plasma membrane repair was shown using an ANX interaction‐defective S100A11 mutant able to rescue repair defects in S100A11 KO cells [53].

Interestingly, ANXA2‐EGFP/S100A10‐mCherry exhibits a FRET efficiency of 13.1% (Fig. 1B), whereas ANXA2‐mCherry/S100A10‐EGFP exhibits a FRET efficiency of only 4.9% (Fig. 8B). Thus, only by swapping the labels the FRET efficiency decreases, which is astonishing, because if C‐termini in ANXA2 and S100A10 complexes were labeled to the same extent, swapping the labels should not have an effect on the FRET efficiencies. The most likely explanation for this discrepancy is that labeled S100A10 can form complexes with other endogenous, unlabeled annexins. As a consequence, S100A10‐EGFP is not always quenched by ANXA2‐mCherry. In contrast to this, EGFP labeled ANXA2 has good chances to bind to a S100A10‐mCherry dimer. In this way two acceptor molecules are brought at the same time into close vicinity of the donor, causing a larger decrease in the fluorescence lifetime. The fact that also in this case unlabeled annexin molecules might interact with S100A10‐mCherry does not affect the measured FRET efficiency, since only donor fluorescence lifetimes are measured.

Interestingly, we detected high FRET efficiencies between labeled S100A11 andANXA2 in cytosolic and cell membrane regions, whereas no fluorescence was detected in nuclei. This is in contrast to S100A11/ANXA1 fluorescence that could be detected in both, cytoplasm and nucleoplasm. Cellular distribution of S100A11 and annexins is possibly influenced by the kind of protein, which is labeled with the fluorescent tag as S100A11 labeled as fluorescence donor appeared beside cytoplasm also in nucleoplasm. However, it is conceivable that S100A11 interaction with distinct annexin members depends on cellular appearance of the interaction partners.

With the FLIM‐FRET approach we analyzed also other potential additional interactions between S100A11 and further annexin family members. By this means, we detected in living cells for the first time a protein complex containing S100A11 and ANXA4. Furthermore, an interaction between S100A11 and ANXA5 could be detected.

Both annexins and S100 proteins are evolutionary conserved protein families [54, 55]. Hence, it is not surprising that a complex comprising rat AnxA6 and human S100A11 was formed.

In summary, by using a fluorescence lifetime‐based FRET measurements we were able to show that S100A11 interacts with several members of the annexin family in living cells. The protein interaction with ANXA2 depends on S100A11´s Ca^2+^‐binding capacity.

## Conflict of interest

The authors declare no conflict of interest.

### Peer review

The peer review history for this article is available at https://www.webofscience.com/api/gateway/wos/peer-review/10.1002/2211-5463.13782.

## Author contributions

CM conceived the project and acquired the data; CM, BH, and CB analyzed and interpreted the data; AW delivered material; CM wrote the manuscript with help of BH and CB.

## Data Availability

The data that support the findings of this study are available from the corresponding author christian.melle@med.uni-jena.de upon reasonable request.

## References

[1] Gerke V and Moss SE (2002) Annexins: from structure to function. Physiol Rev 82, 331–371. 11917092 10.1152/physrev.00030.2001

[2] Miwa N , Uebi T and Kawamura S (2008) S100‐annexin complexes – biology of conditional association. FEBS J 275, 4945–4955. 18795952 10.1111/j.1742-4658.2008.06653.x

[3] Rintala‐Dempsey AC , Rezvanpour A and Shaw GS (2008) S100‐annexin complexes: structural insights. FEBS J 275, 4956–4966. 18795951 10.1111/j.1742-4658.2008.06654.x

[4] Liu Y , Myrvang HK and Dekker LV (2015) Annexin A2 complexes with S100 proteins: structure, function and pharmacological manipulation. Br J Pharmacol 172, 1664–1676. 25303710 10.1111/bph.12978 PMC4376447

[5] Lambert O , Gerke V , Bader MF , Porte F and Brisson A (1997) Structural analysis of junctions formed between lipid membranes and several annexins by cryo‐electron microscopy. J Mol Biol 272, 42–55. 9299336 10.1006/jmbi.1997.1183

[6] Boye TL , Maeda K , Pezeshkian W , Sønder SL , Haeger SC , Gerke V , Simonsen AC and Nylandsted J (2017) Annexin A4 and A6 induce membrane curvature and constriction during cell membrane repair. Nat Commun 8, 1623. 29158488 10.1038/s41467-017-01743-6 PMC5696365

[7] Croissant C , Gounou C , Bouvet F , Tan S and Bouter A (2020) Annexin‐A6 in membrane repair of human skeletal muscle cell: a role in the cap subdomain. Cell 9, 1742. 10.3390/cells9071742 PMC7409186 32708200

[8] Croissant C , Carmeille R , Brevart C and Bouter A (2021) Annexins and membrane repair dysfunctions in muscular dystrophies. Int J Mol Sci 22, 5276. 34067866 10.3390/ijms22105276 PMC8155887

[9] Swairjo MA , Concha NO , Kaetzel MA , Dedman JR and Seaton BA (1995) Ca(2+)‐bridging mechanism and phospholipid head group recognition in the membrane‐binding protein annexin V. Nat Struct Biol 2, 968–974. 7583670 10.1038/nsb1195-968

[10] Swairjo MA and Seaton BA (1994) Annexin structure and membrane interactions: a molecular perspective. Annu Rev Biophys Biomol Struct 23, 193–213. 7522665 10.1146/annurev.bb.23.060194.001205

[11] Jaiswal JK , Lauritzen SP , Scheffer L , Sakaguchi M , Bunkenborg J , Simon SM , Kallunki T , Jäättelä M and Nylandsted J (2015) S100A11 is required for efficient plasma membrane repair and survival of invasive cancer cells. Nat Commun 5, 3795. 10.1038/ncomms4795 PMC4026250 24806074

[12] Koerdt SN and Gerke V (2017) Annexin A2 is involved in Ca^2+^−dependent plasma membrane repair in primary human endothelial cells. Biochim Biophys Acta Mol Cell Res 1864, 1046–1053. 27956131 10.1016/j.bbamcr.2016.12.007

[13] Weisz J and Uversky VN (2020) Zooming into the dark side of human annexin‐S100 complexes: dynamic alliance of flexible partners. Int J Mol Sci 21, 5879. 32824294 10.3390/ijms21165879 PMC7461550

[14] Seemann J , Weber K and Gerke V (1997) Annexin I targets S100C to early endosomes. FEBS Lett 413, 185–190. 9287141 10.1016/s0014-5793(97)00911-3

[15] Santamaria‐Kisiel L , Rintala‐Dempsey AC and Shaw GS (2006) Calcium‐dependent and ‐independent interactions of the S100 protein family. Biochem J 396, 201–214. 16683912 10.1042/BJ20060195 PMC1462724

[16] Rety S , Osterloh D , Arie JP , Tabaries S , Seemann J , Russo‐Marie F , Gerke V and Lewit‐Bentley A (2000) Structural basis of the Ca(2+)‐dependent association between S100C (S100A11) and its target, the N‐terminal part of annexin I. Structure 8, 175–184. 10673436 10.1016/s0969-2126(00)00093-9

[17] Chang N , Sutherland C , Hesse E , Winkfein R , Wiehler WB , Pho M , Veillette C , Li S , Wilson DP , Kiss E *et al*. (2007) Identification of a novel interaction between the Ca(2+)‐binding protein S100A11 and the Ca(2+)‐ and phospholipid‐binding protein annexin A6. Am J Physiol Cell Physiol 292, 1417–1430. 10.1152/ajpcell.00439.2006 17192283

[18] Sakaguchi M , Miyazaki M , Takaishi M , Sakaguchi Y , Makino E , Kataoka N , Yamada H , Namba M and Huh NH (2003) S100C/A11 is a key mediator of Ca^2+^−induced growth inhibition of human epidermal keratinocytes. J Cell Biol 163, 825–835. 14623863 10.1083/jcb.200304017 PMC2173690

[19] Sakaguchi M , Sonegawa H , Murata H , Kitazoe M , Futami JI , Kataoka K , Yamada H and Huh NH (2008) S100A11, a dual mediator for growth regulation of human keratinocytes. Mol Biol Cell 19, 78–85. 17978094 10.1091/mbc.E07-07-0682 PMC2174196

[20] Foertsch F , Teichmann N , Kob R , Hentschel J , Laubscher U and Melle C (2013) S100A11 is involved in the regulation of the stability of cell cycle regulator p21CIP1/WAF1 in human keratinocytes. FEBS J 280, 3840–3853. 23745637 10.1111/febs.12378

[21] Xia Q , Li X , Zhou H , Zheng L and Shi J (2018) S100A11 protects against neuronal cell apoptosis induced by cerebral ischemia via inhibiting the nuclear translocation of annexin A1. Cell Death Dis 9, 657. 29844306 10.1038/s41419-018-0686-7 PMC5974363

[22] Zhao XO , Naka M , Muneyuki M and Tanaka T (2000) Ca(2+)‐dependent inhibition of Actin‐activated myosin ATPase activity by S100C (S100A11), a novel member of the S100 protein family. Biochem Biophys Res Commun 267, 77–79. 10623577 10.1006/bbrc.1999.1918

[23] Murzik U , Hemmerich P , Weidtkamp‐Peters S , Ulbricht T , Bussen W , Hentschel J , von Eggeling F and Melle C (2008) Rad54B targeting to DNA double‐strand break repair sites requires complex formation with S100A11. Mol Biol Cell 19, 2926–2935. 18463164 10.1091/mbc.E07-11-1167 PMC2441681

[24] Foertsch F , Szambowska A , Weise A , Zielinski A , Schlott B , Kraft F , Mrasek K , Borgmann K , Pospiech H , Grosse F *et al*. (2016) S100A11 plays a role in homologous recombination and genome maintenance by influencing the persistence of RAD51 in DNA repair foci. Cell Cycle 15, 2766–2779. 27590262 10.1080/15384101.2016.1220457 PMC5053559

[25] Cecil DL , Johnson K , Rediske J , Lotz M , Schmidt AM and Terkeltaub R (2005) Inflammation‐induced chondrocyte hypertrophy is driven by receptor for advanced glycation end products. J Immunol 175, 8296–8302. 16339570 10.4049/jimmunol.175.12.8296

[26] Guo AJ , Wang FJ , Ji Q , Geng HW , Yan X , Wang LQ , Tie WW , Liu XY , Thorne RF , Liu G *et al*. (2021) Proteome analyses reveal S100A11, S100P, and RBM25 are tumor biomarkers in colorectal cancer. Proteomics Clin Appl 15, e2000056. 33098374 10.1002/prca.202000056

[27] Zeng X , Guo H , Liu Z , Qin Z , Cong Y , Ren N , Zhang Y and Zhang N (2022) S100A11 activates the pentose phosphate pathway to induce malignant biological behavior of pancreatic ductal adenocarcinoma. Cell Death Dis 13, 568. 35752610 10.1038/s41419-022-05004-3 PMC9233679

[28] Sobolewski C , Abegg D , Berthou F , Dolicka D , Calo N , Sempoux C , Fournier M , Maeder C , Ay AS , Clavien PA *et al*. (2020) S100A11/ANXA2 belongs to a tumour suppressor/oncogene network deregulated early with steatosis and involved in inflammation and hepatocellular carcinoma development. Gut 69, 1841–1854. 31919231 10.1136/gutjnl-2019-319019

[29] Gou D , Liu R , Shan X , Deng H , Chen C , Xiang J , Liu Y , Gao Q , Li Z , Huang A *et al*. (2023) Gluconeogenic enzyme PCK1 supports S‐adenosylmethionine biosynthesis and promotes H3K9me3 modification to suppress hepatocellular carcinoma progression. J Clin Invest 133, e161713. 37166978 10.1172/JCI161713 PMC10313362

[30] Memon AA , Sorensen BS , Meldgaard P , Fokdal L , Thykjaer T and Nexo E (2005) Down‐regulation of S100C is associated with bladder cancer progression and poor survival. Clin Cancer Res 11, 606–611. 15701847

[31] Zhang L , Zhu T , Miao H and Liang B (2021) The calcium binding protein S100A11 and its roles in diseases. Front Cell Dev Biol 9, 693262. 34179021 10.3389/fcell.2021.693262 PMC8226020

[32] Clegg RM (1996) Fluorescence resonance energy transfer. In Fluorescence Imaging Spectroscopy Andmicroscopy. *Chemical Analysis Series* 137 ( Wang XF and Herman B , eds), pp. 179–251. Wiley, New York, NY.

[33] Biskup C , Zimmer T , Kelbauskas L , Hoffmann B , Klöcker N , Becker W , Bergmann A and Benndorf K (2007) Multi‐dimensional fluorescence lifetime and FRET measurements. Microsc Res Tech 70, 442–451. 17393489 10.1002/jemt.20431

[34] McCullock TW , MacLean DM and Kammermeier PJ (2020) Comparing the performance of mScarlet‐I, mRuby3, and mCherry as FRET acceptors for mNeonGreen. PLoS One 15, e0219886. 32023253 10.1371/journal.pone.0219886 PMC7001971

[35] Cubells L , Vila de Muga S , Tebar F , Wood P , Evans R , Ingelmo‐Torres M , Calvo M , Gaus K , Pol A , Grewal T *et al*. (2007) Annexin A6‐induced alterations in cholesterol transport and caveolin export from golgi complex. Traffic 8, 1568–1589. 17822395 10.1111/j.1600-0854.2007.00640.x PMC3003291

[36] Jung MJ , Murzik U , Wehder L , Hemmerich P and Melle C (2010) Regulation of cellular actin architecture by S100A10. Exp Cell Res 316, 1234–1240. 20100475 10.1016/j.yexcr.2010.01.022

[37] Koushik SV , Chen H , Thaler C , Puhl HL 3rd and Vogel SS (2006) Cerulean, Venus, and VenusY67C FRET reference standards. Biophys J 91, L99–L101. 17040988 10.1529/biophysj.106.096206 PMC1779932

[38] Dumas JP , Jiang JY , Gates EM , Hoffman BD , Pierce MC and Boustany NN (2019) FRET efficiency measurement in a molecular tension probe with a low‐cost frequency‐domain fluorescence lifetime imaging microscope. J Biomed Opt 24, 1–11. 10.1117/1.JBO.24.12.126501 PMC6935677 31884745

[39] Mailliard WS , Haigler HT and Schlaepfer DD (1996) Calcium‐dependent binding of S100C to the N‐terminal damain of annexin I. J Biol Chem 271, 719–725. 8557678 10.1074/jbc.271.2.719

[40] Hatoum D , Yagoub D , Ahadi A , Nassif NT and McGowan EM (2017) Annexin/S100A protein family regulation through p14ARF‐p53 activation: a role in cell survival and predicting treatment outcomes in breast cancer. PLoS One 12, e0169925. 28068434 10.1371/journal.pone.0169925 PMC5222396

[41] Rintala‐Dempsey AC , Santamaria‐Kisiel L , Liao Y , Lajoie G and Shaw GS (2006) Insights into S100 target specificity examined by a new interaction between S100A11 and annexin A2. Biochemistry 45, 14695–14705. 17144662 10.1021/bi061754e

[42] Albertazzi L , Arosio D , Marchetti L , Ricci F and Beltram F (2009) Quantitative FRET analysis with the EGFP‐mCherry fluorescent protein pair. Photochem Photobiol 85, 287–297. 18764891 10.1111/j.1751-1097.2008.00435.x

[43] Kaufmann T , Herbert S , Hackl B , Besold JM , Schramek C , Gotzmann J , Elsayad K and Slade D (2020) Direct measurement of protein‐protein interactions by FLIM‐FRET at UV laser‐induced DNA damage sites in living cells. Nucleic Acids Res 48, e122. 33053171 10.1093/nar/gkaa859 PMC7708043

[44] Bharadwaj A , Kempster E and Waisman DM (2021) The annexin A2/S100A10 complex: the mutualistic symbiosis of two distinct proteins. Biomolecules 11, 1849. 34944495 10.3390/biom11121849 PMC8699243

[45] Ikebuchi NW and Waisman DM (1990) Calcium‐dependent regulation of actin filament bundling by lipocortin‐85. J Biol Chem 265, 3392–3400. 2137457

[46] Broome AM , Ryan D and Eckert RL (2003) S100 protein subcellular localization during epidermal differentiation and psoriasis. J Histochem Cytochem 51, 675–685. 12704215 10.1177/002215540305100513 PMC3785113

[47] Dempsey AC , Walsh MP and Shaw GS (2003) Unmasking the annexin I interaction from the structure of apo‐S100A11. Structure 11, 887–897. 12842051 10.1016/s0969-2126(03)00126-6

[48] O'Callaghan DW , Ivings L , Weiss JL , Ashby MC , Tepikin AV and Burgoyne RD (2002) Differential use of myristoyl groups on neuronal calcium sensor proteins as a determinant of spatio‐temporal aspects of of Ca^2+^ signal transduction. J Biol Chem 277, 14227–14237. 11836243 10.1074/jbc.M111750200

[49] Bajar BT , Wang ES , Zhang S , Lin MZ and Chu J (2016) A guide to fluorescence protein FRET pairs. Sensors 16, 1488. 27649177 10.3390/s16091488 PMC5038762

[50] Shaner NC , Campbell RE , Steinbach PA , Giepmans BNG , Palmer AE and Tsien RY (2004) Improved monomeric red, orange and yellow fluorescent proteins derived from *Discosoma* sp. red fluorescent protein. Nat Biotechnol 22, 1567–1572. 15558047 10.1038/nbt1037

[51] Gerke V , Creutz CE and Moss SE (2005) Annexins: linking Ca^2+^ signaling to membrane dynamics. Nat Rev Mol Cell Biol 6, 449–461. 15928709 10.1038/nrm1661

[52] Seemann J , Weber K and Gerke V (1996) Structural requirements for annexin I‐S100C complex‐formation. Biochem J 319, 123–129. 8870658 10.1042/bj3190123 PMC1217744

[53] Ashraf APK and Gerke V (2022) The resealing factor S100A11 interacts with annexins and extended synaptotagmin‐1 in the course of plasma membrane wound repair. Front Cell Dev Biol 10, 968164. 36200035 10.3389/fcell.2022.968164 PMC9527316

[54] Morgan RO , Martin‐Almedina S , Iglesias JM , Gonzales‐Flores MI and Fernandez MP (2004) Evolutionary perspective on annexin calcium‐binding domains. Biochim Biophys Acta 1742, 133–140. 15590063 10.1016/j.bbamcr.2004.09.010

[55] Marenholz I , Heizmann CW and Fritz G (2004) S100 proteins in mouse and man: from evolution to function and pathology (including an update of the nomenclature). Biochem Biophys Res Commun 322, 1111–1122. 15336958 10.1016/j.bbrc.2004.07.096

